# A winged helix domain in human MUS81 binds DNA and modulates the endonuclease activity of MUS81 complexes

**DOI:** 10.1093/nar/gkt760

**Published:** 2013-08-27

**Authors:** Andrew J. Fadden, Stephanie Schalbetter, Maureen Bowles, Richard Harris, John Lally, Antony M. Carr, Neil Q. McDonald

**Affiliations:** ^1^Structural Biology Laboratory, Cancer Research UK, London Research Institute, 44 Lincoln’s Inn Fields, London, WC2A 3LY, UK, ^2^Genome Damage and Stability Centre, University of Sussex, Brighton, BN1 9RQ, UK and ^3^Institute of Structural and Molecular Biology, University College London and Birkbeck College, Malet Street, London WC1E 7HX, UK

## Abstract

The MUS81-EME1 endonuclease maintains metazoan genomic integrity by cleaving branched DNA structures that arise during the resolution of recombination intermediates. In humans, MUS81 also forms a poorly characterized complex with EME2. Here, we identify and determine the structure of a winged helix (WH) domain from human MUS81, which binds DNA. WH domain mutations greatly reduce binding of the isolated domain to DNA and impact on incision activity of MUS81-EME1/EME2 complexes. Deletion of the WH domain reduces the endonuclease activity of both MUS81-EME1 and MUS81-EME2 complexes, and incisions made by MUS81-EME2 are made closer to the junction on substrates containing a downstream duplex, such as fork structures and nicked Holliday junctions. WH domain mutation or deletion in *Schizosaccharomyces pombe* phenocopies the DNA-damage sensitivity of strains deleted for *mus81*. Our results indicate an important role for the WH domain in both yeast and human MUS81 complexes.

## INTRODUCTION

The XPF family of eukaryotic DNA junction endonucleases plays crucial roles in maintaining genomic stability by functioning in multiple DNA processing pathways (1). In humans, at least four heterodimeric complexes containing XPF family members have been identified. These include XPF-ERCC1, MUS81-EME1 and the largely uncharacterized MUS81-EME2 complex as well as FANCM-FAAP24, although this complex has no demonstrable nuclease activity to date (1,2). The catalytically active subunits of these complexes have a central nuclease domain distantly related to prokaryotic type II restriction enzymes and two C-terminal helix-hairpin-helix motifs that co-operate as a functional (HhH)_2_ domain to bind DNA (3,4). A similar domain structure is found for the non-catalytic partner of the endonuclease complexes, which are necessary for DNA junction recognition and nucleolytic activity (4–6).

The MUS81 endonuclease, when associated with the non-catalytic partner EME1, is able to efficiently cleave a variety of three- and four-way junctions containing a duplex downstream from a nick *in vitro* (7). These structures include forks, 3′ flaps, nicked Holliday junctions and D-loops (4,8). Such MUS81-EME1 substrates can form during mitosis and fission yeast meiosis and during processing of damaged replication forks.

Structural characterization of MUS81-EME1 has revealed a distinctive orientation of its nuclease and (HhH)_2_ domains and showed an essential contribution from both (HhH)_2_ domains towards DNA junction binding (4). These studies lacked the amino-terminal (N-terminal) extensions of both subunits. Within MUS81, its N-terminal extension contains a single HhH motif, which is believed to be involved in protein–protein interactions rather than DNA binding and has recently shown to be capable of binding Flap endonuclease 1 (FEN1) (9). This study also showed that N-terminal fragments of MUS81 can bind DNA and stimulate the flap endonuclease activity of FEN1. The N-terminal region also interacts with SLX4, a protein that is thought to act as a scaffold for the structure-specific endonucleases MUS81-EME1, XPF-ERCC1 and SLX1 for recruitment to the repair of interstrand crosslinks and restart of damaged replication forks (10–12). The N-terminus was also proposed to contain a Bloom syndrome (BLM) protein-interacting domain (13).

Here, we identify for the first time a winged helix domain (WH domain) within the N-terminal region of human MUS81 that binds DNA, increases the activity of MUS81-EME1/EME2 complexes and influences the incision position of MUS81-EME2 but not MUS81-EME1 complexes on synthetic forks, 3′ flaps and nicked Holliday junctions. Additionally, in MUS81-EME2 complexes, it stimulates the cleavage of splayed arms. Mutations in the WH domain in *Schizosaccharomyces pombe* render a similar sensitivity profile to DNA damaging agents as a *mus81* deleted strain, implying that this domain has a critical role in DNA repair that has been retained in human MUS81.

## MATERIALS AND METHODS

### Protein purification

MUS81-EME1/EME2 complexes (NCBI accession numbers: MUS81,NP_079404.2, EME1: NP_001159603, EME2: NM_001257370) (14) were produced in *Escherichia* coli BL21 (DE3) Rosetta pLysS (Stratagene) using the dicistronic expression plasmid derived from pGEX-KG. MUS81-EME1/EME2 wild-type and mutant complexes were purified as follows: Cultures were grown in Luria-Bertani at 37°C and induced with 25 µM Isopropyl-β-D-thiogalactoside (IPTG) at 18°C overnight. Bacteria were harvested by centrifugation at 4000 × *g* for 15 min at 4°C, and the pellets resuspended in 50 mM sodium phosphate (pH 8), 300 mM NaCl, 2 mM β-mercaptoethanol, 10% glycerol, 0.2% 3-[(3-cholamidopropyl) dimethylammonio]-1-propanesulfonate (CHAPS) (buffer A) supplemented with 10 mM benzamidine, 1 mM phenylmethylsulfonyl fluoride (PMSF) and protease inhibitors (Roche). Bacteria were lysed by sonication. Cell debris was cleared by centrifugation at 29 220 × *g* for 60 min at 4°C, and the clarified lysate was mixed with glutathione-Sepharose 4B (GE Healthcare Lifesciences) for 90 min at 4°C. Unbound proteins were collected, and the affinity resin was washed extensively with buffer A. Human MUS81 (hMUS81) complexes were eluted with elution buffer [buffer A containing 20 mM reduced glutathione (pH 8)]. The eluted proteins were analysed by SDS–PAGE. hMUS81 complexes were concentrated by ultrafiltration and loaded onto a 1 ml of HiTrap Heparin-Sepharose column pre-equilibrated with buffer A. The hMUS81 complexes were eluted using a NaCl gradient (up to 1 M NaCl in buffer A) over 10 column volumes on an AKTA fast performance liquid chromatography system (GE Healthcare Lifesciences). Peak fractions from the Heparin-Sepharose column were pooled and concentrated by ultrafiltration. The concentrated sample was loaded onto a Superose 12 HR 10/300 column (GE Healthcare Lifesciences) pre-equilibrated with buffer A. After analysis by SDS–PAGE, aliquots of complex-containing fractions were flash-frozen in liquid nitrogen for assaying.

#### hMUS81 WH domain purification

Cultures were grown at 37°C and induced by addition of isopropyl-β-D-thiogalactoside (final concentration 250 µM) and further incubation at 37°C for 120 min. Bacteria were harvested by centrifugation at 4000 × *g* for 15 min at 4°C. Cells were resuspended in 50 mM sodium phosphate (pH 7), 500 mM NaCl, 5 mM dithiothreitol (DTT) (buffer A) supplemented with 10 mM benzamidine, 1 mM PMSF and 0.1 mg/ml DNase, and lysed by sonication. Cell debris was cleared by centrifugation at 29 220 × *g* for 30 min at 4°C, and the clarified lysate was mixed with glutathione-Sepharose 4B (GE Healthcare Lifesciences) for 90 min at 4°C. Unbound proteins were collected and the affinity resin was washed extensively with buffer A. hMUS81 WH domain protein was eluted from the affinity resin by addition of GST-tagged 3C protease (PreScission protease, GE Healthcare Lifesciences). hMUS81 WH domain protein was concentrated by ultrafiltration and loaded onto a pre-equilibrated Superdex 75 HR 10/300 column (GE Healthcare Lifesciences) pre-equilibrated with 25 mM sodium phosphate (7), 250 mM NaCl, 1 mM DTT (buffer B). Fractions containing the hMUS81 WH domain monomer were pooled and concentrated by ultrafiltration to ∼10 mg/ml. hMUS81 WH domain proteins for circular dichroism (CD) and DNA binding were grown in Luria-Bertani media. Buffer A was 50 mM Tris (pH 8), 500 mM NaCl, 5 mM DTT and buffer B was 25 mM Tris (8), 250 mM NaCl, 1 mM DTT. The point mutations were made using the Quikchange site directed mutagenesis system (Stratagene). The MUS81 WH deletion mutants were made using overlap PCR to synthesise open reading frames devoid of the WH domain sequence (128–230), and the products were inserted into the dicistronic expression plasmid via the EcoR1 and Xho1 sites. hMUS81 WH domain protein (residues 128–230) was expressed in *E. coli* strain FB810 (BL21 (DE3) recA^-^) from a pET41a plasmid (Novagen). Uniformly ^15^N labelled WH domain protein was produced in minimal medium M9 containing 1 g/l (^15^NH_4_)_2_SO_4_ (Sigma Aldrich) as the nitrogen source. The ^13^C/^15^N labelled WH domain was produced in a similar fashion with ^13^C_6_-D-glucose (Sigma Aldrich) as the only carbon source.

### Nuclear magnetic resonance spectroscopy

Nuclear magnetic resonance (NMR) spectra were acquired at 298 K (except where indicated) on a Varian Unity PLUS spectrometer (operating at a nominal ^1^H frequency of 500 MHz) equipped with a triple resonance probe including a Z-axis pulse field gradient coil. Data acquisition and processing leading to structure calculations are described in Supplementary Information. Atomic coordinates of the final 20 simulated annealing MUS81 conformers and the list of experimental restraints (accession code RCSB 103460) have been deposited at the RCSB Protein Data Bank. Chemical shifts for resonance assignments for the MUS81 have been deposited at the BioMagResBank (accession code 17324).

### Nuclease assays

Oligonucleotides used to make the structures were described previously (15). XO1 and X26 were labelled on the 5′-terminus with [γ32P]. Complementary oligonucleotides were added to the labelled oligonucleotides in two molar excess to form the structures and purified on a 10% non-denaturing polyacrylamide gel in TBE. Reactions were carried out at 25°C for 90 min in reaction buffer containing 5′-^32^P-labelled substrate in 60 mM sodium phosphate (pH 7.4), 5 mM MgCl_2_, 1 mM DTT and 100 µg/ml bovine serum albumin (BSA) in a total volume of 20 µl. Incision products were separated on a sequencing gel (Sequagel, National Diagnostics) for 2 h. The gel was removed and dried, and products were visualized by auroradiography, or a STORM phosphorimager (Molecular Dynamics). Quantification of data was carried out using ImageQuant TL software (GE healthcare Life Sciences).

#### Fluorescence anisotropy

Fluorescence polarization anisotropy experiments were carried out as described previously (14).

### *In vivo* analysis in *S. pombe*

A *mus81* base strain was constructed in *S.pombe*, which is compatible for recombination-mediated cassette exchange (16). The open reading frame of *mus81* as well as 160 bp of upstream sequence was replaced with the ura4^+^ gene flanked by loxP and loxM3 sites. Wild type and mutant *mus81* constructs were re-introduced into the endogenous locus by recombination-mediated cassette exchange using loxP- and loxM3-flanked *mus81* on a Cre-recombinase expression plasmid. This allows expression of mutants and wild-type at the native locus by the *mus81* promoter. The point mutations were introduced by site-directed mutagenesis. A WH domain deletion mutant, *mus81*-*ΔWH*, was constructed by fusion PCR by combining residues 1–116 and 215–608, and therefore eliminating residues 117–214. All *mus81* variants were confirmed by sequencing after integration.

For DNA damage-sensitivity assays, cells were grown at 30°C overnight in yeast extract (YE) media, serially diluted and spotted onto agar plates starting with 10^5^ cells. YEA plates containing methyl methanesulfonate (Fluka 64294), camptothecin (98%, Acros Organics 7689-03-4), 4-nitroquinoline 1-oxide (Fluka 73265), Hydroxyurea (98% Sigma H8627) at the indicated concentrations were used. Plates were incubated for 4 days at 30°C.

Whole cell protein extracts were prepared by TCA precipitation. For the chromatin fractionation assay, chromatin was separated by centrifugation through a sucrose cushion as in Liang and Stillman 1997 (17) except the buffer for cell wall digestion with Zymolyase and lysing enzymes was 50 mM sodium citrate, 40 mM EDTA, 1.2 M sorbitol.

## RESULTS

### Identification of an uncharacterized amino-terminal domain within human MUS81

**Figure 1.**
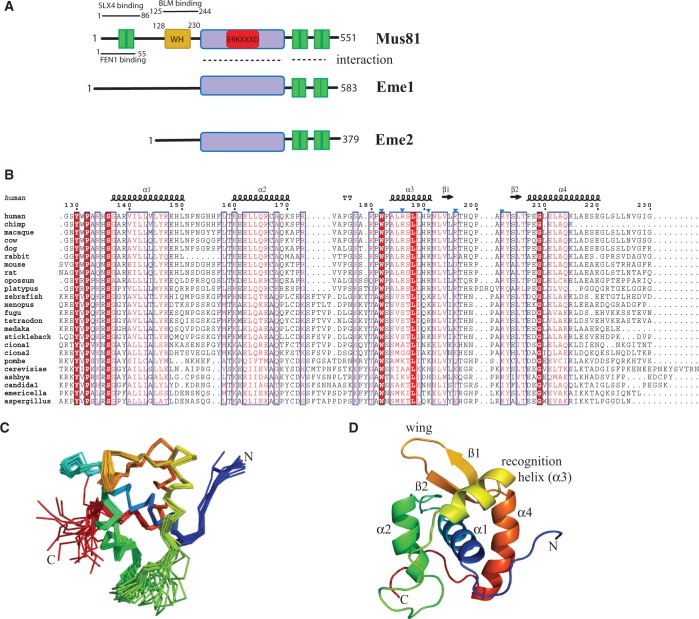
Human MUS81 contains a WH domain. (**A**) Domain structure of human MUS81 and its partners EME1 and EME2. The red box indicates the catalytic motif within the nuclease domain (mauve). HhH motifs are shown in green. Regions involved in known protein–protein interactions are indicated. (**B**) Structure-based sequence alignment of selected eukaryotic MUS81 WH domains; secondary structures above the sequences are from the NMR structure. Mutated residues in human helix α3 are indicated by blue triangles. (**C**) Ensemble of NMR structures of WH domain. The domain comprises four helices (residues 137–150, 160–169, 183–191, 207–220) and two ß strands (residues 194–197 and 202–205) (**D**) A representative WH domain structure indicating the location of the recognition helix and wing motif.

The major portion of the human MUS81 (Accession code: NP_079404.3) amino-terminus has few characterized domains (Figure 1A). Our sequence alignments and structure predictions identified a conserved region of ∼100 amino acids (residues 128–230 from human MUS81) present in most eukaryotic MUS81 orthologues excepting nematode and fruit fly species (Figure 1B*).* Analysis of this region by the Phyre recognition server (18) gave a best fold prediction with an *E*-value of 0.34 as a WH tertiary fold. A recombinant form of residues 128–230 from human MUS81 was prepared and characterized (see Supplementary Methods). Size exclusion chromatography and CD revealed the protein migrated as an 11.5 kDa protein and exhibited CD spectra consistent with a predominantly helical secondary structure (data not shown).

### Structure of the hMUS81 WH domain

**Figure 2.**
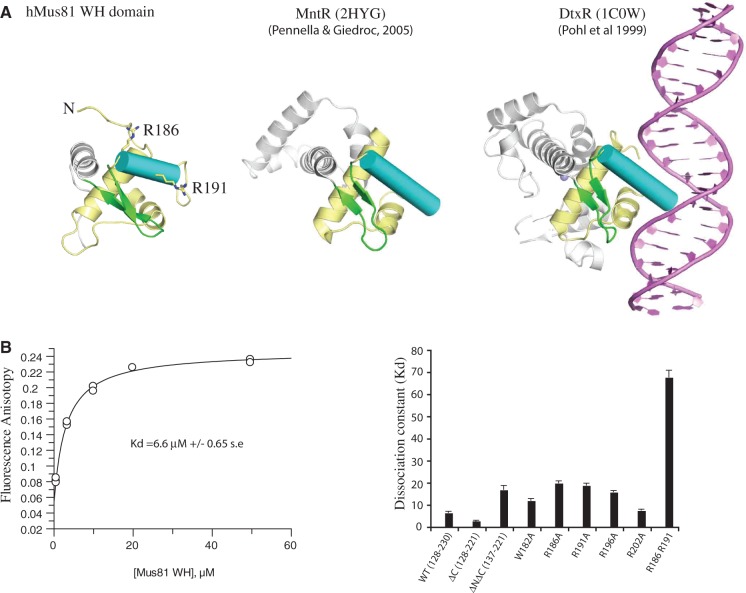
The hMUS81 WH domain recognition helix binds DNA. (**A**) Comparison of close structural relatives of hMUS81 WH domain with selected members of the DtxR repressor family. The recognition helix (cyan cylinder) and wing motif (green ß-strands) are shown with selected basic residues represented as sticks. DtxR is bound to dsDNA in the canonical WH domain DNA-binding mode. (**B**) Dissociation constants (K_d_) obtained for hMUS81 WH domain and selected mutants binding to duplex DNA (units µM, s.e. = standard error). Values were obtained from fluorescence anisotropy curves using 50 nM DNA and purified hMUS81 WH domains.

The 3D structure of residues 128–230 from hMUS81 was then determined by heteronuclear NMR (Figure 1C and Supplementary Table S1). The structure revealed a helical domain that adopts a WH tertiary fold (Figure 1D), commonly found in a large number of DNA-binding proteins (19). The hMUS81 WH domain contains two functionally important elements of WH proteins, namely, the recognition helix α3 and the ß-hairpin ‘wing’ motif (Figure 1D). The short wing motif of hMUS81 is well ordered even in the absence of DNA. Strikingly, the recognition helix (helix α3) is at least two turns shorter than many other WH domain recognition helices, and its amino-terminal end is highly mobile, despite the presence of several prolines and a buried W182 sidechain. Sequences immediately preceding the recognition helix (helix α2/α loop) segregate into two clade-specific clusters: those belonging to higher vertebrate MUS81 and those of lower vertebrates and eukaryotes (Figure 1B). Many MUS81 invariant residues are buried including three that precede helix α1 (Y130, P132 and S146; Figure 1B), suggesting the angle between the amino-terminus and helix α1 is important. The hMUS81 WH domain is highly basic (pI 10.0) with 12 Arg/Lys sidechains that are distributed throughout the sequence. These include two arginines within the recognition helix (R186 and R191 as discussed later), suggesting a possible charge complementarity appropriate for binding DNA (Figure 2A).

Structural comparisons with the hMUS81 WH domain indicate the closest similarity is to the manganese-binding MnTR (Z-score of 9.1 and rmsd of 2.1 Å over 66 C-alpha atoms), a metal-dependent bacterial repressor protein belonging to a DtxR-like family of transcriptional regulators (20,21) (Supplementary Figure S1, Figure 2A). These WH proteins bind DNA using a canonical DNA interaction seen previously (e.g. HNF-3γ and E2F) involving major groove recognition by helix α3 and phosphodiester binding by one of the wing motifs (22). The similarity to hMUS81 WH domain strongly suggests it that too is capable of a similar DNA-binding function. Analysis of this structural similarity revealed a short motif T-X-X-G-X-Hy-A/G (Hy = hydrophobic residue) within helix α4 that, together with preceding hydrophobic residues in the recognition helix α3, provides a sensitive fingerprint for identifying closely related WH proteins to hMUS81 (Supplementary Figure S1). In hMUS81, T206 acts as an N-cap to helix α4, hydrogen bonding to mainchain amides and also across to a carbonyl of the helix α3-ß1 loop, similar to the metal-dependent transcriptional regulator WH domains. The WH motif also includes L205 and G209 from hMUS81 that play important structural roles at the amino-end of helix α4 and contact conserved buried residues from helix α1. A213 is also frequently a small sidechain and packs against Y146 sidechain of helix α1.

### The hMUS81 WH domain binds DNA

Residues 125–244 of MUS81 were previously reported to bind to the RecQ helicase homologue, BLM (13). However, we were unable to demonstrate this interaction using purified recombinant proteins (Fadden, data not shown). The close similarity to transcriptional repressors strongly suggests that the hMUS81 WH domain would use a similar duplex DNA-binding mechanism. NMR chemical shift titration with N^15^-labelled MUS81 WH domain and duplex DNA shifted residues within regions 131–142 (amino-terminus and helix a1) and 186–191 (recognition helix α3 including R186, L188 and R191), thus implicating these regions in DNA binding (Supplementary Figure S2A and B).

The contribution of different regions of the WH domain to DNA binding was measured by fluorescence polarization (Figure 2B). Carboxy-terminal truncations to the WH domain resulted in a slight increase in DNA binding (ΔC; 128–221) (Figure 2B). However, truncation of both termini reduced DNA binding (NC; 137–221) dramatically. Together, these results suggest a role for both the amino-terminus and the recognition helix of the WH domain in binding DNA.

To establish whether MUS81 WH domain is a major groove binding canonical WH domain, individual mutations within helix α3 (W182A, R186A, R191A, R196A and R202A) were prepared. These mutations markedly reduced DNA binding (Figure 2B). Combining R186A and R191A mutations from the recognition helix increased the dissociation constant ∼10-fold strongly implicating helix α3 as a crucial element that engages double-stranded DNA in a similar manner to canonical WH domains.

### The WH domain modulates the incision activity of MUS81 complexes

**Figure 3.**
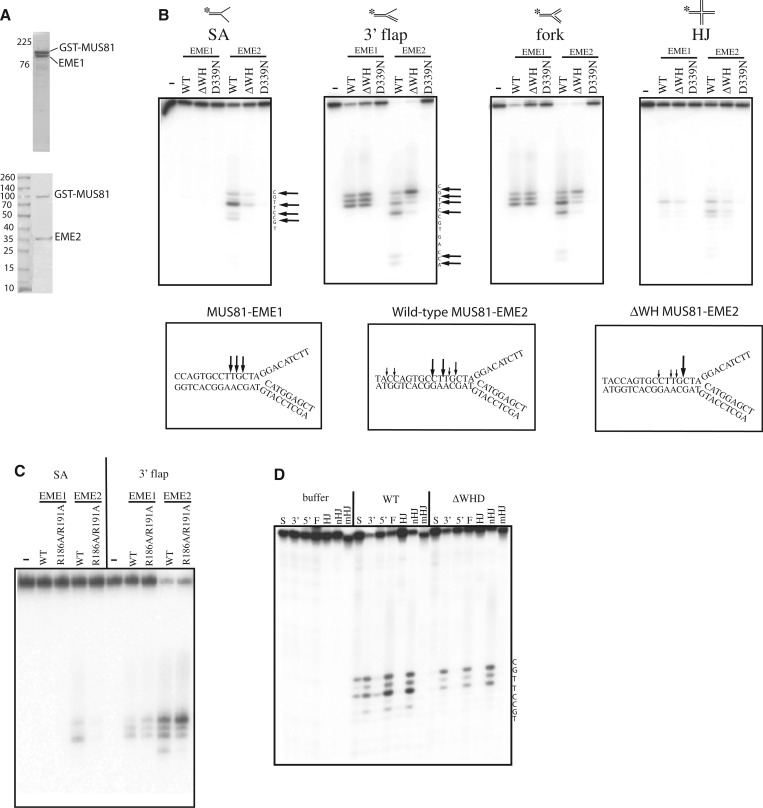
The WH domain modulates the endonuclease activity of MUS81 complexes. (**A**) Effect of WH domain on incisions by MUS81 complexes. (A) SDS–PAGE gels showing purified wild-type (WT) MUS81-EME1 and MUS81-EME2 proteins. The anomalous migration pattern of EME1 and has been observed before (2). (**B**) Sequencing gels showing products of incision reaction by 25 fmol purified recombinant MUS81-EME1/2 wild-type or ΔWH domain complexes, and the MUS81 D339N dead mutant on 75 fmol substrates as indicated, labelled with ^32^P (asterisk). (−) is protein buffer only. SA is splayed arm; HJ, Holliday junction; Arrows show the positions of the incisions made. Schematic below shows the cleavage pattern of MUS81 at the junction on model 3′ flap complexes. Large and small arrows indicate the relative intensities of the incisions made by the MUS81 complexes on the 3′ flap. (**C**) Sequencing gel showing products of incision reaction by purified recombinant MUS81-EME2 wild-type or WD domain α3 helix R186/191A mutants on 3′ flaps and forks. (**D**) Sequencing gels showing incision sites of MUS81-EME2 and MUS81ΔWH-EME2 on various substrates. S is splayed arm, 3′ is 3′ flap, 5′ is 5′ flap, F is fork, HJ is Holliday junction, nHJ is nicked Holliday junction, mHJ is mobile Holliday junction.

To study the effect of the WH domain of MUS81 on incision activity, we prepared recombinant MUS81-EME1 and MUS81-EME2 complexes and compared the endonuclease activity of 25 fmol of each on splayed arm, 3′-flap, fork and Holliday junctions, (Figure 3A). The catalytically impaired MUS81 D339N mutation (23,24) inactivated both MUS81-EME1 and MUS81-EME2 complexes (Figure 3A) confirming that the nuclease activity was specific. To compare the effects of removal of the WH domain on the endonuclease activity of MUS81-EME1/EME2 complexes, the data for splayed arm, 3′ flap and fork substrates from Figure 3A were quantified by densitometry, and the proportion of each band calculated as a percentage of the total present in the lane (Supplementary Table S2). In general, MUS81-EME2 complexes converted more of its substrates to products than the same concentration of MUS81-EME1, suggesting that MUS81-EME2 is intrinsically more active (Figure 3A and Supplementary Table S2). This is in agreement with another study (14). Furthermore, both complexes cleaved substrates with a duplex downstream from a junction, in preference to splayed arms. Under the conditions of these experiments, which contained 25 fmol of protein complexes and 75 fmol of substrate, MUS81-EME1 complex was unable to cleave the splayed arm (Figure 3A and Supplementary Table S2), whereas the same amount of MUS81-EME2 converted nearly half of it into incision products (Figure 3A and Supplementary Table S2). Deletion of residues 128–230 corresponding to the WH domain from MUS81-EME2 reduced total cleavage of the splayed arm substrate, and the incisions occurred at the same position as wild-type (Figure 3A and Supplementary Table S2).

On both 3′ flaps and forks MUS81-EME1 made three incisions of similar intensity upstream from the junction and deletion of the WH domain resulted in decreased total incision activity (Supplementary Table S2). This contrasts with previous studies where it was shown that removal of 246 amino acids from the N-terminus of MUS81 did not impact on the incision activity of recombinant MUS81-EME1 complexes (4,25), although the EME1 used in these studies was derived from a shorter transcript variant (26). More than 95% of the 3′ flap and fork substrate is cleaved by wild-type MUS81-EME2 under these conditions, and deletion of the WH domain reduced this to 89 and 82% for the 3′-flap and fork, respectively. MUS81-EME2 makes incisions further upstream from the junction than MUS81-EME1 (Figure 3A) on these substrates, and the nicked Holliday junction (Figure 3C). Deletion of the WH domain results in movement of the dominant incision site closer to the junction and indicates that stabilization of DNA binding of the complex by the WH domain of MUS81 has a role in positioning the incision sites in MUS81-EME2. Both MUS81-EME1 and MUS81-EME2 cleaved intact Holliday junctions less well, as observed previously for MUS81-EME1 (Figure 3A) (4), and there was no cleavage of single-stranded DNA (data not shown). The MUS81 R186A/R191A double mutation within the helix α3 of the WH domain, which reduced the DNA-binding affinity (Figure 2B), also showed a similar incision pattern to the WH deletion mutant (Figure 3C). These data imply that DNA binding by the WH domain in MUS81 increases the activity of both MUS81 complexes *in vitro* and allows incisions to be made further from the junction by MUS81-EME2 complexes on 3′ flaps, forks and nicked Holliday junctions.

The substrate preferences of MUS81-EME2 have not been previously characterized so we tested the incision activity of MUS81-EME2 and the MUS81ΔWH domain deletion mutant on a range of substrates that included a mobile Holliday junction (X26) containing 26 centrally placed complementary base pairs allowing the junction to migrate. This structure is thought to be more representative of an *in vivo* migratable Holliday junction and was shown previously to be weakly cleaved by purified MUS81-EME1 (2). Here, we show that WT MUS81-EME2 did not cleave the mobile HJ and instead preferred to cleave substrates with a duplex downstream from a nick, or discontinuity. Surprisingly, fewer incisions were made on a 5′ flap, indicating that its upstream duplex may be inhibiting binding of MUS81-EME2 to the downstream single strand.

### Role of the WH domain *in vivo*

**Figure 4.**
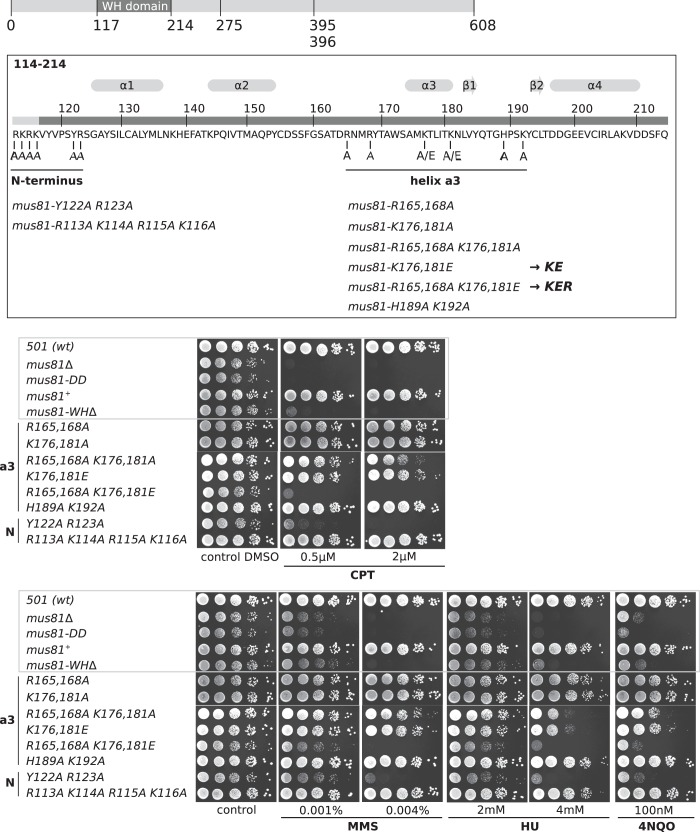
Mutations in the WH domain of *S. pombe mus81* phenocopies a *mus81* deletion. Top panel shows the *S. pombe* WH domain sequence and structural motifs predicted from the alignment in Figure 1. The mutated residues are indicated. Bottom panel shows spot tests on agar plates containing DNA damaging agents. CPT, campothecin; MMS, methylmethanesulfonate; HU, hydroxyurea; 4NQO, 4-Nitroquinoline 1-oxide.

The structural similarities to repressors, the WH domain DNA-binding properties and effects on the endonucleolytic incision combined provide compelling evidence that in human MUS81, the WH domain of MUS81 binds duplex DNA. Human EME1 and EME2 are both distant homologues of *S. pombe* Eme1, although human EME2 is much shorter (2). To address the role of the WH domain *in vivo*, we mutated *S. pombe mus81* while keeping the gene expressed by its own promoter at the native locus. WH domain deletion, and the double point mutation D395A/D396A (a catalytically dead form of *mus81*) (27) reduced *S. pombe* viability slightly, and conferred similar levels of sensitivity to chronic exposure to DNA-damaging agents as deletion of the entire *mus81* gene (Figure 4). N-terminal substitutions in regions implicated in DNA binding by the human WH domain were tested. Mutation of the tetra-basic motif RKRK (residues 113–116) to alanines did not sensitize the cells to DNA damage. However, mutating YR at 122–123 in the N-terminal region directly adjacent to helix α1 increased sensitivity to all DNA-damaging agents to levels similar to that of *mus81-ΔWHD*. The double mutants R165A/R168A and H189A/K192A located just outside the region of helix α3 had no effect on the DNA damage sensitivity. Replacement of K176 and K181 [corresponding to R186 and R191 in human (Figure 1)] with alanine also results in no change in sensitivity. However, substitution of K176 and K181 (KE) with glutamate, which enhances the effect of losing the basic sidechains, resulted in an intermediate sensitivity to HU and 4NQO, but not to CPT or MMS. This suggests that these residues are involved in processing a subset of structures produced during the repair of lesions induced by these agents. The KE mutant is proficient in meiosis with a spore viability similar to wild-type in contrast to *mus81* and *mus81-ΔWHD*, which displayed spore viabilities of <1% of wild-type (data not shown). Combining the K176A/K181A double mutation with R165A/R168A (KER) results in a moderate increase in sensitivity to HU and 4-NQO and a similar phenotype to *mus81-ΔWH*. These results show that the WH domain of Mus81 is crucial for resistance to DNA-damaging agents in *S. pombe* and implies that this *in vivo* function has been retained in mammals.

### WH mutations do not affect protein localization

**Figure 5.**
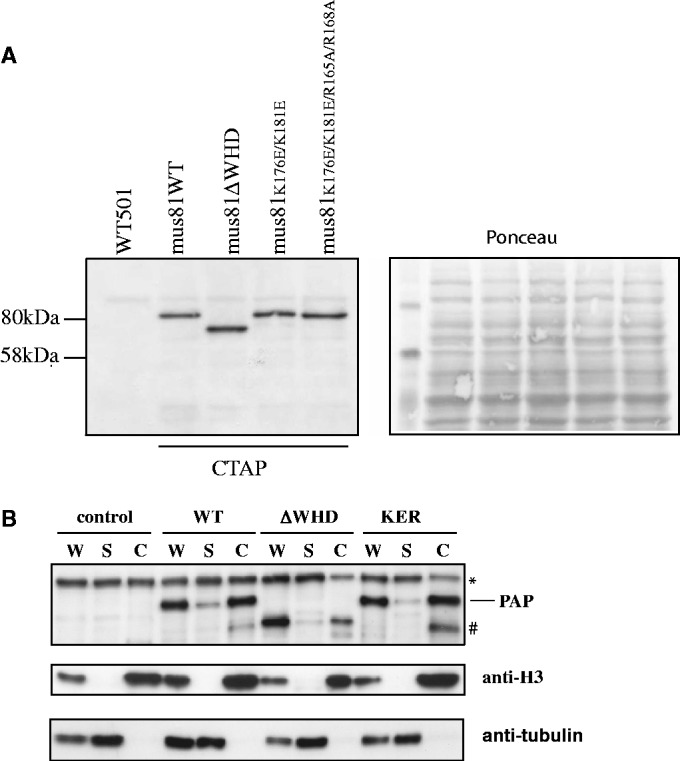
Mutations in the WH domain do not affect cellular localization of Mus81-Eme1. (**A**) Top panel: Immunoblot of whole-cell extracts from cells expressing C-terminally TAP-tagged Mus81 wild-type and mutant proteins. Bottom panel: Ponceau staining of the immunoblot to show protein loading. TAP-tagged proteins were detected with peroxidase anti-peroxidase (PAP). The first lane contains extract from an untagged control strain (501). (**B**) Chromatin fractionation of TAP-tagged Mus81+, Mus81-ΔWH and Mus81-KER. W; whole extract, S; soluble fraction/supernatant, C; chromatin fraction. Samples were separated by SDS–PAGE. Control lanes contain an untagged wild-type strain. The Mus81-TAP constructs were detected with peroxidase anti-peroxidase (PAP). The arrow indicates the TAP-tagged proteins, (asterisk) is a non-specific band and (hash) is a presumed degradation product.

The N-terminus of Mus81 was shown to be required for nuclear localisation in *Saccharomyces cerevisiae* (28). We therefore investigated the localization of *mus81*-*WH* mutant complexes in *S. pombe* cells by chromatin fractionation. A C-terminal TAP tag was fused to the endogenous copy of the *mus81* mutants (Figure 5A). TAP-tagged wild-type, Mus81-ΔWH, Mus81-KE and Mus81-KER proteins were expressed at their expected sizes and at similar levels (Figure 5A). The tagged strains did not have a significant phenotype. For the chromatin fractionation, exponentially growing cells were harvested and the cell wall digested. After lysis, the whole cell extracts (W) were separated using sucrose cushion into soluble (S) and chromatin fraction (C) (Figure 5B). The fractions of wild-type and mutant C-terminally TAP-tagged strains were analysed by western blot (Figure 5B). The TAP-tagged Mus81, Mus81-ΔWH and Mus81-KER proteins each accumulated in the chromatin fraction. However, the level of Mus81-ΔWH was reduced in the chromatin fraction compared with Mus81 and Mus81-KER, but it is unlikely that this would cause the observed phenotype, as the Mus81-KER mutant with a similar phenotype shows levels comparable with wild-type Mus81 in the chromatin fraction. We conclude that the phenotype of the *mus81-WH* mutants was not due to mislocalization of the proteins in the cell.

## DISCUSSION

### A role for the WH domain in MUS81-EME2 complexes

We report the identification, 3D structure and both *in vitro* and *in vivo* characterization of a previously unnoticed WH domain within MUS81. In human recombinant MUS81, this domain appears to enhance the incision activity of both MUS81-EME1 and MUS81-EME2 complexes, and modulate the positioning of MUS81-EME2 incisions on 3′ flaps, forks and nicked Holliday junctions.

Our results for MUS81-EME1 contrasts with studies where the endonuclease activity of the complex was not affected by the removal of the N-termini of both MUS81 and EME1 (4,25,26). There are currently two accepted transcript variants of EME1, which have been reported to display different levels of activity when recombinant forms of the MUS81-EME1 were expressed and immunoprecipitated from Hela cells (26), although these had additional single amino acid additions and substitutions. We have used the longer EME1_583_ transcript variant of EME1, isolated by Ciccia *et al.* (2), which has a 13 amino acid insert after residue 371 residing within the 36R linker region (residues 368–403) identified by Chang *et al.* (4) and shown to be important for DNA binding and endonuclease activity. Their experiments suggest that lengthening the 36R linker by replacing it with the equivalent region in zebrafish reduced the endonuclease and DNA-binding activity of recombinant N-terminally truncated MUS81-EME1_570_ complexes, and it was postulated that MUS81-EME1_583_ might be less active because of the length of the insert. In our experiments, the 13 amino acids insert might inhibit binding of the 36R region of EME1 to DNA in such a way that binding of the WH domain to duplex DNA becomes dominant and would explain why removal of the WH domain decreases the activity in the MUS81-EME1_583_ complex. Detailed kinetic comparisons of recombinant versions of both EME1 transcript variants will be required to ascertain the contribution of the WH domain to the endonuclease activity of each MUS81-EME1 complex.

The WH domain influences the positioning of the incision site made by MUS81-EME2 on substrates such as 3′ flaps, forks and nicked Holliday junctions that contain an upstream and downstream duplex and deletion of the WH domain consistently moves the major incision site towards the junction by up to five bases (Figure 3, Supplementary Table S2). This is probably a result of enhanced bending or opening of the duplex at the junction and given that the incision pattern is exactly the same on all three of these substrates, we conclude that the WH domain must bind duplex DNA or interact with subdomains in the MUS81-EME2 complex or both. Alternatively, it is possible that it may contribute to substrate recognition by stable binding of the downstream duplex as well as the MUS81 (HhH)_2_ domain, as modelled by Chang *et al.* (4).

The splayed arm substrate lacks a downstream duplex, and we find that in our assays the WH domain increases cleavage activity on splayed arms by MUS81-EME2. The activity of the MUS81-EME1 complexes is weaker for all the substrates, and cleavage of the splayed arm was not detectable at the concentrations used. N-terminally deleted recombinant human MUS81-EME1_570_ complexes have been shown to be capable of cleaving splayed arms at high concentrations (100 nM); (4) therefore, it is likely that the MUS81 (HhH)_2_ domain or other components of the C-terminal complex can interact weakly with the downstream single strand of the splayed arm in MUS81-EME1. It is conceivable that the WH domain may stabilize a weaker interaction of the C-terminal domains with the downstream single strand in the splayed arm.

**Figure 6.**
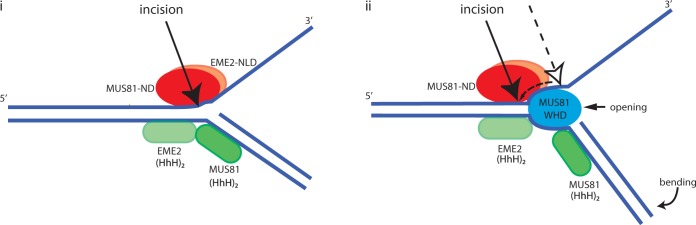
Model for the interaction of recombinant MUS81-EME2 with synthetic substrates. (i) Substrates such as 3′-flaps, replication forks and nicked Holliday junctions contain a downstream duplex, which is bound by the (HhH)_2_ domain of MUS81 causing conformational changes which trigger cleavage. For splayed arms, this interaction is weak and not detectable without the MUS81-WH domain. (ii) The WH domain of MUS81 plausably binds the upstream duplex near or at the junction and, together with interaction with other domains, bends the downstream duplex and/or unwinds the upstream duplex, thus moving the incision position further from the junction. MUS81-ND is MUS81 nuclease domain; EME2-NLD is EME2 nuclease-like domain; MUS81WHD is MUS81 WH domain. On splayed arms, the interaction of the MUS81-WH domain described above may stabilize the weak binding of the MUS81-EME2 C-terminal domains sufficiently to facilitate cleavage, but is not tight enough to allow opening/bending required to move the incision position.

A hypothetical model for the interaction of MUS81-EME2 with a fork is shown in Figure 6. We note that cleavage of the 5′ flap by MUS81-EME2 is poor, and the WH domain reduces this activity. We speculate that the presence of a second duplex with inappropriate polarity results in a non-productive interaction that blocks substrate incision. The N-terminal truncated human MUS81-EME1 used in (4) was unable to cleave 5′ flaps even at the same concentration (100 nM) that produced incisions on a splayed arm, indicating that the mechanism of inhibition by the second duplex does not involve the WH domain.

### Implications for the activity of MUS81 complexes in the cell

The N-terminus of hMUS81 containing the WH domain is well conserved in *S. pombe*, and we found that the WH domain is essential for both meiosis and DNA damage tolerance in this organism. The role of the Mus81WH domain in meiosis in *S. pombe* suggests that its DNA binding activity is necessary for the processing of intermediates such as D loops or Holliday junctions in the absence of a Yen1/GEN1 resolvase homologue (29). We found here that point mutations in two residues (K176E and K181E) of the WH domain predicted to be involved in DNA binding have no effect on meiosis but show a different drug sensitivity profile to WH domain deletion mutants implying that there may be a separation of function, which is manifested as recognition and cleavage of different structures during replication fork repair. Further examination of this finding is necessary but beyond the scope of this study. Enhanced bending or opening of the DNA at the junction of the structures by MUS81-EME2 may yield products with larger single strand gaps and affect their downstream processing possibly indicating a link between the different drug sensitivities of the KE mutants in *S. pombe* and the role of the WH domain in MUS81-EME2 activity.

We note that duplex DNA binding by N-terminal fragments of MUS81 has been suggested to stimulate FEN1 cleavage of double flap substrates (9,30). Such an interaction may link the WH domain of MUS81-EME1/EME2 complexes to Okazaki fragment processing during stalled replication fork repair.

Overall, our data suggest that EME1 gene duplication in metazoan genomes may have resulted in differences in processing of the substrates cleaved by MUS81. Further structural and biochemical analyses are evidently required to reveal the basis for DNA junction recognition and cleavage by full-length MUS81-EME complexes containing the WH domain.

## ACCESSION NUMBERS

Coordinates have been deposited at the RCSB protein data bank as RCSB ID [rscb103460] and PDB ID code [2mc3].

## SUPPLEMENTARY DATA

Supplementary Data are available at NAR Online, including [31–40].

## FUNDING

Funding for open access charge: Cancer Research UK core funding to the London Research Institute (to N.Q.M.); MRC grant [G1100074 to A.M.C.].

*Conflict of interest statement.* None declared.

## Supplementary Material

Supplementary Data
