# Supplementary material for: A winged helix domain in human MUS81 binds DNA and modulates the endonuclease activity of MUS81 complexes
Source: Nucleic Acids Res. 2013 Aug 27;41(21):9741–52. doi: 10.1093/nar/gkt760 (PMC3834828; doi:10.1093/nar/gkt760)
Supplement: Supplementary Data [file supp_41_21_9741__index.html]

A winged helix domain in human MUS81 binds DNA and modulates the endonuclease activity of MUS81 complexes — Supplementary Data 

# A winged helix domain in human MUS81 binds DNA and modulates the endonuclease activity of MUS81 complexes

## Supplementary Data

files

**Files in this Data Supplement:**

- Supplementary Data - pdf file
- Supplementary Data - pdf file
