# Supplementary figures and images for: A winged helix domain in human MUS81 binds DNA and modulates the endonuclease activity of MUS81 complexes
Source: Nucleic Acids Res. 2013 Aug 27;41(21):9741–52. doi: 10.1093/nar/gkt760 (PMC3834828; doi:10.1093/nar/gkt760)

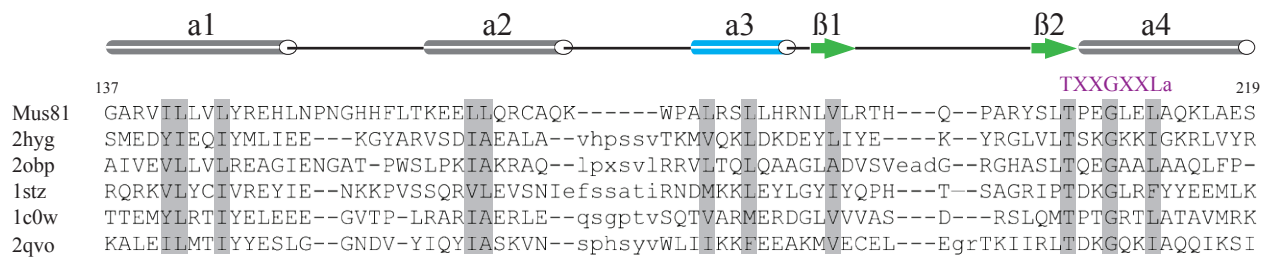

Figure S1

Fadden et al.

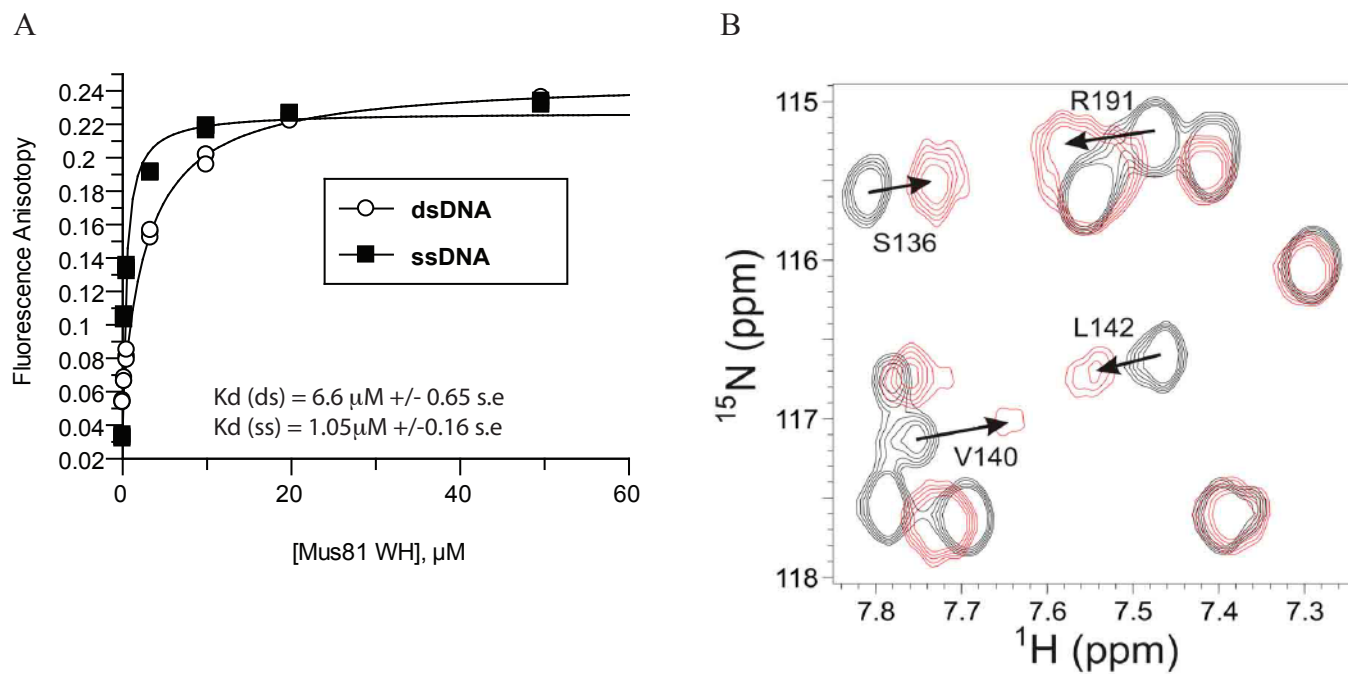

Figure S2

Fadden et al.

Supplement: Supplementary Data [file supp_gkt760_nar-01948-m-2012-File008.pdf]
