# Supplementary material for: A winged helix domain in human MUS81 binds DNA and modulates the endonuclease activity of MUS81 complexes
Source: Nucleic Acids Res. 2013 Aug 27;41(21):9741–52. doi: 10.1093/nar/gkt760 (PMC3834828; doi:10.1093/nar/gkt760)
Supplement: Supplementary Data [file supp_gkt760_nar-01948-m-2012-File009.pdf]

SUPPLEMENTARY MATERIAL

Table S1: Summary of NMR structure statistics for hMus81 WH domain

Experimental restraints ii	<SA>	SAlowest
All (Å) (1628)	0.056 ± 0.002	0.054
Intraresidue (498)	0.044 ± 0.004	0.040
Sequential (386)	0.060 ± 0.004	0.061
Short (354)	0.058 ± 0.003	0.052
Long (383)	0.063 ± 0.004	0.062
Ambiguous (7)	0.020 ± 0.009	0.016
Hydrogen bond restraints (Å) (38)	0.082 ± 0.012	0.077
Dihedral angle restraints (°) (110)	0.91 ± 0.15	0.735
Residual dipolar couplings (Hz) (71)	0.17 ± 0.02	0.17
Number of residual restraints violations		
NOE violations > 0.5 Å	0.25 ± 0.4	0
Angle violations > 5°	1.4 ± 0.9	0
RDC violations > 0.5Hz	0.15 ± 0.4	0
Deviations from idealised covalent geometry iii		
Bonds (Å) (1886)	0.0023 ± 0.0001	0.0023
Angles (°) (3414)	0.53 ± 0.01	0.51
Improper dihedrals (°) (998)	0.3 ± 0.01	0.4
Structural statistics for the ensemble iv		
PROCHECK parameters		
Most favoured region (%)	78.4 ± 3.1	77.9
Additionally allowed (%)	18.9 ± 0.26	20.9
Generously allowed (%)	1.4 ± 1.3	0.0
Disallowed (%)	1.3 ± 0.5	1.2
Number of bad contacts	6 ± 2	3
R-factor for residual dipolar coupling restraints (%)	0.8 ± 0.1	0.8
RMSD from the average structure v		
Backbone (N, Cα, C) (Å)	0.44 ± 0.06	0.48
Heavy atoms (Å)	0.86 ± 0.09	0.85

<SA>, represents the set of 20 selected conformers obtained by restrained dynamical simulated annealing in CNS. SAlowest refers to the lowest energy structure of the set.

ii Sum averaging of NOE distance restraints was used for groups with degenerate proton chemical shifts. The interproton unambiguous distance restraint list comprised 498

intraresidue, 386 sequential (|i – j|= 1|), 354 short range (1<|i – j|<5), and 383 long-range (|i – j|>5). Hydrogen-bonds restraints were applied as pairs of distance restraints: HN•••O, 1.2-2.2 Å; N•••O, 1.2-3.2 Å. The final values for the respective force constants were: NOE, 30 kcal mol-1 Å-2; H-bonds, 50 kcal mol-1 Å-2; dihedral angles, 200 kcal mol-1 rad-2.

iii The final values for the respective force constants were: bond lengths, 1000 kcal mol-1 Å-2; angles and improper torsions, 500 kcal mol-1 rad–2; the improper torsion angle restraints serve to maintain planarity and chirality.

iv The program PROCHECK (Laskowski et al., 1993) was used to assess the stereochemical parameters of the family of conformers for the VirB9 moiety only. The figures indicate the percentage of residues with backbone ϕ and ψ angles in separate regions of the Ramachandran plot, defined in the program. The number of bad contacts per 100 residues is expected to be in the range 0-30 for protein crystal structures of better than 3.0Å resolution.

v The precision of the atomic coordinates is defined as the average pairwise RMSD between each of the 20 conformers and a mean coordinate structure SA generated by iterative best-fit of the backbone atoms (N, Cα, and C) over residues 3-45 and 55-98 of Mus81 (comprising the core secondary structure elements and omitting the flexible N and C termini and the disordered loop between α2 and α3) followed by coordinate averaging.

Table S2: Quantification of data showing the endonuclease activity of Mus81 complexes in Figure 3A

	Splayed arm				3'-flap				Fork			
	Mus81-Eme1		Mus81-Eme2		Mus81-Eme1		Mus81-Eme2		Mus81-Eme1		Mus81-Eme2	
	WT	ΔWH	WT	ΔWH	WT	ΔWH	WT	ΔWH	WT	ΔWH	WT	ΔWH
uncleaved	100	100	53.5	85.5	42	52.5	4.7	11.3	33.1	64.4	4.4	17.8
A-T												
T-C												
C-G			7.2	3.7	12.7	15.1	14.7	71.7	8.5	8.0	10.4	47.8
G-T			0.49		17.9	13.8	1.7	8.1	21.7	10.2	10.2	9.0
T-T			31.9	10.6	26.5	18.6	20.1	7.7	36.3	17.4	42	17.6
T-C												
C-C			2.3				40	0.4			24	7.8
C-G			5.6				10.1					

Numbers represent the amount of cleavage product as a % of the total in each lane on the autoradiograph in Fig 3A. The positions of the incisions in the duplex upstream from the junction are shown in the first column, and represent the phosphodiester bond between the 2 bases (shown in Fig 3)

Supplementary Figures

Figure S1: Structure-based alignment of the hMus81 WH domain with the WH domain from selected DtxR-like repressor proteins obtained by DALI (Holm & Sander, 1995). A conserved sequence fingerprint at the C-terminus of the WH domain is indicated as well as the recognition helix and wing motif.

Figure S2: A) 1H – 15N HSQC spectra for the DNA titration experiment. A short DNA duplex was titrated into a solution of 15N-labelled hMus81 WH domain. B) Selected chemical shifts are shown.

Supplementary Methods

NMR spectroscopy. Sequence-specific resonance assignments were obtained using standard triple resonance NMR spectroscopy. Distance restraints were derived from 3D 15N- and 13C-edited NOESY-HSQC spectra with a mixing time of 120ms and 100ms, respectively. One-bond 1H-15N residual dipolar couplings were obtained from [1H,15N]IPAP-HSQC spectra [1] acquired at 291K in the presence and absence of 5% n-octyl-penta(ethylene glycol):octanol, 0.96:1 [2]. NMR spectra were processed using NMRpipe/NMRDraw [3] and analyzed using ANSIG for openGL v1.0.3[4] 1H, 13C, and 15N chemical shifts were referenced indirectly to sodium 2,2-dimethyl-2-silanepentane-5-sulfonate (DSS), using absolute frequency ratios for the 1H signals [5].

Structure calculations. Interproton distance restraints were derived from the ANSIG cross peaks file of 3D 15N-NOESY-HSQC and 13C-NOESY-HSQC spectra. Most resonances were manually assigned with the remaining cross-peaks assigned using through-bond correlation spectra and the "Connect" module from the program AZARA [6]. All structures for hMus81 WH domain were calculated using an ab initio simulated annealing protocol within the CNS program [7] with PARALLHDGv5.3 force field and PROLSQ non-bonded energy function [8]. A total of 1628 NOE-derived interproton distance restraints for hMus81 WH domain were included in the final iterations of the structure calculations (see Table 1). Backbone torsion angle restraints for f and y were derived from analysis of 1Ha, 13Ca, 13Cb, 13C', and 15NH chemical shift databases as implemented in the program TALOS [9]. Hydrogen bond restraints for amide protons were derived from an assessment of the regular secondary structure elements. This analysis included the overall and local patterns of NOEs and the pattern of amide proton solvent exchange rates. A total of 110 dihedral angle and 38 hydrogen bond (19 H-bonds; 2 distance restraints per H-bond) interatomic distance restraints were used for hMus81 WH domain.

Initial estimates of the axial and rhombic components of the molecular alignment tensor were obtained using the program MODULE-1 [10] using the lowest energy structure determined without residual dipolar couplings, 13.8Hz and 0.68Hz, respectively. These values were used as a starting point for a procedure that simultaneously refined the protein structure and ascertained the values of the alignment tensor components using a grid search approach. The final values of the axial component and rhombicity of the molecular alignment tensor used in the refinement were 14.9 Hz and 0.67Hz, respectively. A total of 71 RDC restraints were used for hMus81 WH domain.

SUPPLEMENTARY REFERENCES

1. Ottiger, M., F. Delaglio, and A. Bax, Measurement of J and dipolar couplings from simplified two-dimensional NMR spectra. J Magn Reson, 1998. **131**(2): p. 373-8.
2. Ruckert, M. and G. Otting, Alignment of Biological Macromolecules in Novel Nonionic Liquid Crystalline Media for NMR Experiments. J. Am. Chem. Soc. , 2000. **122**: p. 7793-7797.
3. Delaglio, F., et al., NMRPipe: a multidimensional spectral processing system based on UNIX pipes. J Biomol NMR, 1995. **6**(3): p. 277-93.
4. Kraulis, P.J., ANSIG: A program for the assignment of protein 1H2D NMR spectra by interactive graphics. . J. Magn. Reson. , 1989. **24**: p. 627-633.
5. Wishart, D.S., et al., 1H, 13C and 15N random coil NMR chemical shifts of the common amino acids. I. Investigations of nearest-neighbor effects. J Biomol NMR, 1995. **5**(1): p. 67-81.
6. Boucher, W., AZARA v2.0 Department of Biochemistry, University of Cambridge, UK., 1996.
7. Brunger, A.T., et al., Crystallography & NMR system: A new software suite for macromolecular structure determination. Acta Crystallogr D Biol Crystallogr, 1998. **54**(Pt 5): p. 905-21.
8. Linge, P.J. and M. Nilges, Influence of non-bonded parameters on the quality of NMR structures: a new force field for NMR structure calculation. . J. Biomol. NMR, 1999. **13**: p. 51-59.
9. Cornilescu, G., F. Delaglio, and A. Bax, Protein backbone angle restraints from searching a database for chemical shift and sequence homology. J Biomol NMR, 1999. **13**(3): p. 289-302.
10. Dosset, P., et al., A novel interactive tool for rigid-body modeling of multi-domain macromolecules using residual dipolar couplings. J Biomol NMR, 2001. **20**(3): p. 223-31.
